# Prevalence and clinical features of respiratory syncytial virus in children hospitalized for community-acquired pneumonia in northern Brazil

**DOI:** 10.1186/1471-2334-12-119

**Published:** 2012-05-16

**Authors:** Letícia Martins Lamarão, Francisco Luzio Ramos, Wyller Alencar Mello, Mirleide Cordeiro Santos, Luana Soares Barbagelata, Maria Cleonice Aguiar Justino, Alexandre Ferreira da Silva, AnaJudithPiresGarcia Quaresma, Veronilce Borges da Silva, Rommel Rodríguez Burbano, Alexandre Costa Linhares

**Affiliations:** 1 Instituto de Ciências Biológicas, Universidade Federal do Pará, Avenida Augusto Correa 01, 66075-900, Belém, PA, Brazil; 2 Instituto Evandro Chagas, Secretaria de Vigilância em Saúde, Ministério da Saúde, Belém, PA, Brazil; 3 Hospital Universitário João de Barros Barreto, Universidade Federal do Pará, Belém, PA, Brazil

## Abstract

**Background:**

Childhood pneumonia and bronchiolitis is a leading cause of illness and death in young children worldwide with Respiratory Syncytial Virus (RSV) as the main viral cause. RSV has been associated with annual respiratory disease outbreaks and bacterial co-infection has also been reported. This study is the first RSV epidemiological study in young children hospitalized with community-acquired pneumonia (CAP) in Belém city, Pará (Northern Brazil).

**Methods:**

With the objective of determining the prevalence of RSV infection and evaluating the patients’ clinical and epidemiological features, we conducted a prospective study across eight hospitals from November 2006 to October 2007. In this study, 1,050 nasopharyngeal aspirate samples were obtained from hospitalized children up to the age of three years with CAP, and tested for RSV antigen by direct immunofluorescence assay and by Reverse Transcription Polymerase Chain Reaction (RT-PCR) for RSV Group identification.

**Results:**

RSV infection was detected in 243 (23.1%) children. The mean age of the RSV-positive group was lower than the RSV-negative group (12.1 months vs 15.5 months, *p*<0.001) whereas gender distribution was similar. The RSV-positive group showed lower means of C-reactive protein (CRP) in comparison to the RSV-negative group (15.3 vs 24.0 mg/dL, *p*<0.05). Radiological findings showed that 54.2% of RSV-positive group and 50.3% of RSV-negative group had interstitial infiltrate. Bacterial infection was identified predominantly in the RSV-positive group (10% vs 4.5%, p<0.05). Rhinorrhea and nasal obstruction were predominantly observed in the RSV-positive group. A co-circulation of RSV Groups A and B was identified, with a predominance of Group B (209/227). Multivariate analysis revealed that age under 1 year (*p*<0.015), CRP levels under 48 mg/dL (*p*<0.001) and bacterial co-infection (*p*<0.032) were independently associated with the presence of RSV and, in the analyze of symptoms, nasal obstruction were independently associated with RSV-positive group (*p*<0.001).

**Conclusion:**

The present study highlights the relevance of RSV infection in hospitalized cases of CAP in our region; our findings warrant the conduct of further investigations which can help design strategies for controlling the disease.

## Background

Globally, RSV is the most common cause of childhood acute lower respiratory infection and is responsible for annual outbreaks worldwide [1-4]. RSV infection usually results in upper respiratory tract illness characterized by profuse rhinorhea, however 25 – 40% of children experiencing infections in their first year of life may develop severe respiratory disease requiring hospitalization [1,5,6]. This may result in long-term respiratory disorders such as abnormal pulmonary function, asthma, recurrent cough, and bronchitis [7,8]. RSV has two Groups, A and B, which are distinguished largely by antigenic and genetic characteristics. During epidemics, either Group A or B may predominate, or both Groups may circulate concurrently [7,9]. Evidence for RSV infection has been found in every geographic area studied and the predominant occurrence changes according to the region’s climates. In temperate countries RSV outbreaks coincide with winter and in tropical climates the pattern varying with most literature associating RSV with rainy season [10-13].

Pneumonia is among the main causes of illness and death in the younger children throughout the world [14-16]. There is the need for a better assessment of the epidemiology of viral CAP in developing countries where RSV infections substantially account for epidemics and are associated with a more severe clinical presentation of pneumonia [4]. Some retrospective studies investigated the occurrence of bacterial coinfection in children hospitalized with severe RSV infection and found the incidence of pulmonary bacterial coinfection to vary between 17.5 and 44% [15,17,18].

To our knowledge, this is the first RSV epidemiological study in children hospitalized with CAP in Belém city, Pará, Northern Brazil, to assess the epidemiological, clinical, and laboratory features of RSV infections among infants and young children. Moreover we sought to characterize the circulating RSV Groups in our region.

## Methods

### Study population

Patients’ clinical and epidemiological data were obtained during a cross-sectional study comprising eight hospitals carried out between November 2006 and October 2007, in Belém, Pará, located in the Northern tropical area of Brazil. We investigated for RSV infection in hospitalized children with CAP. This condition was as defined by medical practitioners of the project based on (A), (B) and (C). (A) Two clinical findings: cough, history of fever, pleuritic pain, crackles or bronchial breath sounds; (B) Chest radiographic findings consistent with pneumonia (focal airspace consolidation, patchy increased interstitial markings), and (C) patients admitted with less than 48 hours. The last condition was used to exclude patients had been infected with pneumonia after being admitted to the hospital. Patients were included in this study if the following criteria were met: children up to the three years old, hospitalized, diagnosed with CAP and with a signed consent form obtained from parents or legal guardians at enrolment. This study was approved by the Ethics and Research Committee of Evandro Chagas Institute (IEC) in the context of a large prospective study that investigates the etiology of CAP in different countries.

### Sample collection

Nasopharyngeal aspirate samples were collected by vacuum suction through a plastic catheter and refrigerated at 4°C until transported on ice to the Respiratory Virus Laboratory at IEC. At the institute, the samples were processed within two hours for RSV antigen detection using the Direct Immunofluorescence Assay (DFA). RSV-positive samples were subsequently subjected to RT-PCR for the detection of the RSV Group.

Demographic data and clinical symptoms were also obtained. A questionnaire was filled by a trained technician including age, onset of symptoms, gender, blood bacterial culture, chest radiography, C-Reactive Protein (CRP) levels, and signs or symptoms of cough, rhinorrhea, fever, nasal obstruction, vomiting and diarrhea were analyzed at the time of hospitalization.

### Direct immunofluorescence assay (DFA)

DFA was carried out using specific monoclonal antibodies for the detection and identification of RSV in direct respiratory specimen cell preparation with Light Diagnostics^TM^ Respiratory Syncytial Virus DFA kit, cat. 3125 (Chemicon® International, Inc. Temecula, CA), in accordance with the manufacturer’s instructions.

### Reverse transcriptase-polymerase chain reaction (RT-PCR)

RT-PCR was performed to detect RSV RNA sequence and identify RSV Groups in samples that were positive by DFA. Primers were adopted from Canducci et al. [19]. Total RNA was extracted directly from supernatant specimens with the QIAamp® Viral RNA Mini Kit, cat. 52904 (Qiagen) and the amplification and detection were performed with SuperScript^TM^ One-step RT-PCR and the Platinum *Taq*® comercial Kit, cat. 10928–034 (Invitrogen Life Technologies, CA, USA). The primers used for group determination were generated against the F regions of the RSV genome. Positive control and water as a negative control were run together in each RT-PCR assay to validate the amplification process and to exclude the presence of contaminants.

### C-reactive protein (CRP)

CRP levels were determined from serum samples of patients and stored up to 4 days at about 4°C, until the completion of qualitative and semi-quantitative survey, by agglutination of latex particles using the Serolatex PCR kit, cat. 56 (Labtest Diagnóstica S.A., Brazil), in accordance with the manufacturer’s instructions.

### Radiographs

The radiological assessment was performed by three independent physicians blinded to the patient’s condition, with reading of the radiographs standardized for all. Radiological typical for CAP included interstitial infiltrate, alveolar infiltrate, and lobar pneumonia.

### Statistical analyses

Patients were divided into two groups according to DFA results and the analysis was performed using Statistical Package for the Social Sciences (SPSS, version 17.0, Inc., Chicago, IL), for Windows. Fisher’s exact test was used adjunctively if the expected values were less than 5. The student’s *t* test was applied to compare means.

Multivariate analysis was performed using logistic regression models. Variables with a *p* value <0.1 in univariate analysis were entered in the multivariate analysis. The level of significance was set at <0.05. The both analyses (1 and 2) including pneumonia positive and negative for RSV as the dependent variable. (1) was a comparison of pneumonia by RSV positive and negative group included the following independents variables: age, gender, CRP levels and bacterial culture; (2) was a comparison of pneumonia positive and negative for RSV and the signs and symptoms reported at hospitalization included the following variables: cough, rhinorrhea, fever, nasal obstruction, vomiting and diarrhea.

## Results

The inclusion criteria were met by 1,214 patients with CAP during the period of study, of whom 1,050 (86.49%) had consent from parents or legal guardians to participate. The differences in distribution of demographic characteristics and admission diagnoses were not statistically significant for those who declined relative to those who participate in the study (data not shown). RSV DFA results were available for all 1,050 patients included in the study. The prevalence of RSV infection was 23.1% (243/1,050 patients). Demographic and clinical features of RSV- positive and RSV-negative children are shown in Table 1. In terms of age, the average of RSV-positive group (12.1 months) was lower than that of the RSV-negative group (15.5 months) (*p*<0.001). There was no statistically significant difference between the groups in relation to gender (51.8% male and 48.2% female). Among the patients who had CRP levels analyzed (810/1.050, 77.1%), the RSV-positive group showed a lower mean level in comparison to the RSV-negative group (*p*<0.001). The chest radiological findings have shown that 54.2% of RSV-positive and 50.3% of RSV-negative patients developed interstitial infiltrate. Bacterial culture were available for 46.7% of study participants (490/1.050). Although the 90.0% of RSV-positive patients yielded negative bacteriological culture, bacterial co-infection was identified in this group with 10.0% of culture growth, while the RSV-negative group showed only 4.5% growth (*p*<0.05) (Table 1).

**Table 1:** Epidemiologic, clinical, and laboratory characteristics of RSV-positive and RSV-negative children hospitalized for community-acquired pneumonia in Belém, Para, Brazil

**Characteristics**	**RSV- positive 243 (23.1%)**		**RSV-negative 807 (76.9%)**			**Groups (n)**		
	**n**	**%**	**n**	**%**	** *p* **	**A**	**B**	** *p* **
**Age (years)**								
0 - 1	152/241	63.1	340/805	42.1	**<0.001**	10/18	135/207	0.282
**Gender**								
Male	126/243	51.8	449/807	55.6	0.113	9/18	108/207	0.542
**C-Reactive protein**								
≤48 mg/dL	155/178 87.0		469/632 74.2		**<0.001**	9/13	133/151	**<0.001**
**Radiographs**								
Interstitial infiltrate	117	54.2	366	50.3	0.085	9	102	0.235
Alveolar infiltrate	49	22.7	164	22.5		1	45	
Lobar pneumonia	50	23.1	198	27.2		5	39	
**Bacterial culture**								
Positive	11^**Δ**^/109	10.0	17^*^/381	04.5	**0.043**	1/8	10/92	0.342

^**Δ**^*S. epidermidis* (3 patients), *A. baumannii* (2), *S. pneumoniae* (1), *Com. acidovorans* (1), *K. pneumoniae* (1), Gram Positive (1), *Candida spp* (1), other fungi (1).

* *S. pneumoniae* (4 patients), *S. epidermidis* (3), Tetrads (3), Gram Positive (3), *K. pneumoniae* (2), *S. viridians* (1), *A. baumannii* (1).

It was possible to determine the RSV Group in 227 (93.4%) out of 243 RSV-positive samples. RSV Group B infections predominated RSV Group A (209 vs 18 patients, respectively). Group B infection was associated with a lower age than Group A (11.0 vs 13.0 months; *p*<0.03). With regards to the CRP levels, Group B infection showed a lower CRP mean when compared to Group A (11.0 vs 19.0 mg/dL, *p*<0.05). Gender, radiological pattern, bacterial culture and the onset of symptoms denoted a similar distribution in both Groups.

We described the signs and symptoms in Table 2 for RSV-positive and RSV-negative groups. Approximately 98% of RSV-positive children had a cough at admission but no statistically significant difference was observed when compared to the RSV-negative group (96.1%; *p*>0.05). Five clinical parameters showed significantly different rates (*p*<0.05) when comparing both groups: fever, vomiting and diarrhea were detected predominantly in the RSV-negative group (80.2% vs 72.4%, 12.2% vs 4.9% and 8.1% vs 4.1%, respectively), while rhinorrhea and nasal obstruction were predominantly observed in RSV-positive group (78.2% vs 71.5% and 59.2% vs 32.8%, respectively, both comparisons yielding a *p*<0.05). Patients infected with either A or B Groups did not significantly differ in terms of signs and symptoms.

**Table 2:** Signs and symptoms reported at hospitalization in RSV-positive and RSV-negative patients with community-acquired pneumonia and detected RSV Groups

	**RSV-positive 243 (23.1%)**		**RSV-negative 807 (76.9%)**			**Groups**		
	**n**	**%**	**n**	**%**	** *p* **	**A**	**B**	** *p* **
Cough	238	97.9	714	96.1	0.056	100%	98.5%	0.779
Rhinorrhea	190	78.2	531	71.5	**0.002**	83.3%	77.8%	0.443
Fever	176	72.4	596	80.2	**0.009**	83.3%	71.8%	0.323
Nasal obstruction	144	59.2	244	32.8	**<0.001**	66.6%	58.7%	0.359
Vomiting	12	04.9	91	12.2	**<0.001**	0	4.8%	0.428
Diarrhea	10	04.1	60	08.1	**0.023**	0	14.8%	0.428

**Figure 1:**
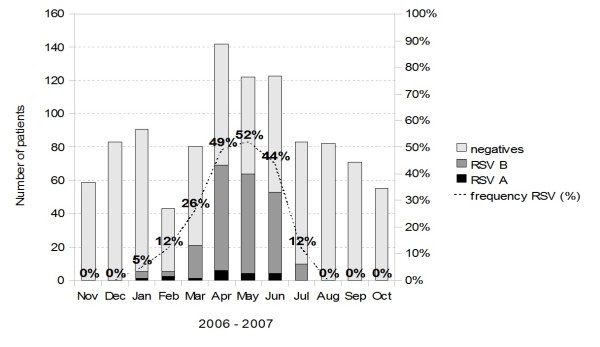
Monthly prevalence of Respiratory Syncytial Virus (RSV) in children up to three years old, hospitalized with community-acquired pneumonia (CAP), from November 2006 to October 2007.

Figure 1 shows that RSV infection was detected from January to July 2007, with higher prevalence rates observed from April (48.6%) to June 2007 (52.4%).

The Multivariate Analysis of Risk Factors for RSV CAP showed in analyze (1) the age under 1 year (OR 1.36; 95% CI; 1.06–1.74; *p*<0.015), CRP levels under 48 mg/dL (OR 3.49; 95% CI; 1.80–6.77; *p*<0.001) and bacterial co-infection (OR 2.57; 95% CI; 1.08–6.09; *p*<0.032) were independently associated with the presence of RSV as opposed to RSV-negative group. In analyze (2) only nasal obstruction was independently associated with presence of RSV (OR 3.07; 95% CI; 2.24–4.21; *p*<0.001) (Table 3).

**Table 3:** The Multivariate Analysis of Risk Factors for RSV infection in patients hospitalized with community-acquired pneumonia

**Analyzes**		** *p* **	**OR**	**95% CI**	
				**Lower**	**Upper**
**Signs and symptoms**					
	Fever	0.109	0.754	0.535	1.065
	Rhinorrhea	0.798	0.952	0.654	1.386
(1)	Nasal obstruction	<0.001	3.075	2.244	4.215
	Cough	0.107	2.428	0.825	7.146
	Diarrhea	0.646	0.836	0.390	1.794
	Vomiting	0.078	0.551	0.283	1.069
**Characteristics**					
	CRP <48 mg/dL	<0.001	3.498	1.807	6.772
(2)	Bacterial co-infection	0.032	2.570	1.081	6.094
	Age <1 year	0.015	1.361	1.062	1.745
	Male gender	0.476	1.186	0.742	1.895

(1) (2) pneumonia positive and negative for RSV as the dependent variable.

## Discussion

The detection of RSV in 23.1% of the samples in our study denoted a prevalence rate similar to those observed in other studies on the occurrence of lower respiratory tract infection, ranging from 23% to 61% [6,20,21]. The possible limitation of the study was the use of a less sensitive method (IF) as compared to RT-PCR that has shown improved sensitivity in the detection of RSV infection [14,22]. In fact there is differential detection related to viral load. Infants usually have severe RSV disease associated with higher viral load hence a better chance of detection compared to the older counterparts leading to a differential misclassification (BIAS) [23]. However, the large number of samples analyzed in this study minimizes the bias and did not alter the statistical significance of our results.

RSV has been referred in the literature as the main agent responsible for bronchiolitis and pneumonia during the first year of life. According to our study, 62.5% of children were younger than one year old, suggesting that illnesses caused by RSV can be severe in this age group, thus requiring hospitalization with prompt and effective medical intervention [5,24].

Gender was not identified as a significant risk factor for RSV infection in our study in agreement with other published studies [21,25]. Nevertheless, some findings in the literature have shown a male predominance, as reported by D’Elia et al. [5], particularly between 0 and 2 months.

Evaluation of CRP is very useful for clinicians because it may help differentiate between bacterial and viral etiologies. Shin et al. [26] used levels of CRP (≥1.87 mg/dL) as criteria to rule out serious bacterial infection in infants from self-limiting viral illness in febrile infants younger than three months. Diniz et al. [25] also found a statistically significant difference between nosocomial viral lower respiratory tract infection and levels of CRP less than or equal to 40 mg/L. In our study, the RSV-positive group showed a lower CRP mean level when compared to the RSV-negative group. Thus, low CRP levels may suggest a viral infection and, depending upon the clinical and radiological findings, enables the suspension of antibiotic therapy and helps considerably in the reduction of the hospital stay.

The radiological findings in our study have shown that 54.2% of RSV-positive patients developed interstitial infiltrate. Diniz et al. [25] found a significant correlation between nosocomial viral lower respiratory tract infection and interstitial infiltrate and it was observed that all patients with confirmed bacterial, fungal, or mixed infection presented alveolar infiltrate.

We found a predominance of RSV infection without bacterial co-infection in our study, in agreement with Duttweiler et al. [27] who found that concomitant bacterial sepsis was a rare event in 127 hospitalized RSV infected infants. However, when we compared the groups, positive culture was predominantly observed in the RSV-positive group than the RSV-negative group (10.0% vs 4.5%, *p*<0.028). Thorburn et al. [15] reported on pulmonary bacterial co-infection in children with severe RSV bronchiolitis showing that 40% of children with severe RSV infection were infected with bacteria in their lower airways. Unfortunately, we were unable to demonstrate if these infections would be either secondary or concurrent to viral infection.

It was possible to determine the RSV Group in 227 (93.4%) samples, and 6.6% were untypable probably due to problems in processing samples or RNA extraction. This study reports predominance of RSV Group B infection in children hospitalized with CAP, which is not unusual in other regions [9,28]. Unfortunately, our data presents some limitation since we could collect samples for only one year and annual RSV Group epidemic may change. Suwanjutha et al. [28] identified in the first year of study the predominance of Group B, in contrast to the second year when Group A was more predominant.

Due to the high number of RSV Group B infection, comparison with.Group A (only 18 children) epidemiological data is not conclusive. However, in our findings, Group B was found among children with a mean age lower than that for Group A (11.0 vs 13.0; *p*<0.03), a finding similar to that of Papadopoulos et al. [29] who reported a predominance of RSV B infection in the youngest children, but the reasons are still unknown. Mlinaric-Galinovic et al. [9] and Oliveira et al. [8] did not find age differences between Groups, even though Mlinaric-Galinovic et al. [9] found that Group B infections occurred more frequently in males less than 12 months of age than in females. Oliveira et al. [8] on the other hand, found that the 57.8% of RSV A-infected children were male. Unfortunately, there is no plausible explanation for this variation.

We were able to determine that the CRP mean level in Group B was lower than Group A (13.0 vs 19.0; *p*<0.05) but the reasons for this remain unknown. Further research with an adequate number of samples needs to be conducted in an attempt to better understand RSV Groups in cases of CAP and their association with the epidemiological data of patients.

We have evaluated the signs and symptoms and approximately 98% of RSV-positive cases had cough at admission, but no statistically significant difference was observed in comparison with RSV-negative group (96.1%) (*p*>0.05). Rhinorrhea and nasal obstruction were predominantly observed in the RSV-positive group (*p*<0.05). Regarding the clinical signs and symptoms observed by Diniz et al. [25] in São Paulo city, in the preterm infants infected with RSV, wheezing, rhinorrhea, vomiting, and diarrhea were significantly more frequent while in our study vomiting and diarrhea were detected predominantly in RSV-negative group. In addition, the clinical symptoms do not predict the viral etiology because there are difficulties in establishing the general etiologic diagnoses of pneumonia by clinical profiles, which are quite varied in literature and depend on the infectious agent as well as the age and immune state of the host.

With regards to the seasonality, our study showed that RSV activity in hospitalized children during January to July 2007, with a peak during April to June, coincided with a heavy rainfall period in the region. Outbreaks occurring mainly during the months with low temperatures were reported in Uberlandia (Midwestern Brazil) by Costa et al. [30]. This seasonal pattern was also observed in other Brazilian settings where RSV occurrence is not uniform. This has been noted in several Brazilian geographic regions and even among different states in the same region [10]. In Campinas (São Paulo) [21] and in Vitória (Espírito Santo) [31], South-eastern region, the annual highest incidence of RSV-infections occurred between January and June, the same period of months observed in our study.

A greater understanding of the factors that determine RSV activity would make this timing even more precise. However, such studies are important as they are expected to delineate the clinical and epidemiological behavior of RSV in this age range and in this region. Even though our study presents a low prevalence of bacterial co-infection among the RSV-positive patients, monitoring RSV activity is necessary in order to restrict antibiotic use to the infants in real need and to provide better prophylactic therapies.

## Conclusion

In conclusion, to our knowledge this is the first report of RSV infection in hospitalized children with CAP in our region. We highlighted that the RSV infection prevalence associated with CAP in Northern Brazil is similar to those found in other regions and other countries. However, according to current findings and those reported in the literature, it may be concluded that the lower mean of age as well as the lower levels of CRP are characteristics found in viral infections of these cases in Belém city. The signs and symptoms associated with RSV in our study were rhinorrhea and nasal obstruction, even though divergent in the literature, and the predominant RSV Group identified was B. Furthermore, the data provided by the study indicate that there is a need for continuous efforts in order to broaden our knowledge on the epidemiological aspects of RSV infection and CAP.

## Abbreviations

RSV, Respiratory syncytial virus; CAP, community-acquired pneumonia; RT-PCR, Reverse Transcription Polymerase Chain Reaction; IEC, Evandro Chagas Institute; DFA, Direct Immunofluorescence Assay; CRP, C-Reactive Protein; CI, Confidence interval.

## Competing interest

The authors declare that they have no competing interests.

## Authors’ contributions

LML, FLR, WAM and ACL designed the study. LML, AFS, FLR, MCAJ, AJPGQ and VBS collected the data. LML, MSC and LSB performed the technique. LML, WAM, ACL made the interpretation of statistical analyses. LML, WAM, RRB and ACL wrote the paper with input from all the authors who each approved the final version.

## Pre-publication history

The pre-publication history for this paper can be accessed here:

http://www.biomedcentral.com/1471-2334/12/119/prepub
